# Thorium Removal, Recovery and Recycling: A Membrane Challenge for Urban Mining

**DOI:** 10.3390/membranes13090765

**Published:** 2023-08-29

**Authors:** Geani Teodor Man, Paul Constantin Albu, Aurelia Cristina Nechifor, Alexandra Raluca Grosu, Szidonia-Katalin Tanczos, Vlad-Alexandru Grosu, Mihail-Răzvan Ioan, Gheorghe Nechifor

**Affiliations:** 1 Analytical Chemistry and Environmental Engineering Department, University Politehnica of Bucharest, 011061 Bucharest, Romania; man_geani@yahoo.com (G.T.M.); aureliacristinanechifor@gmail.com (A.C.N.); andra.grosu@upb.ro (A.R.G.); 2 National Research and Development Institute for Cryogenics and Isotopic Technologies—ICSI, 240050 Râmnicu Valcea, Romania; 3 Radioisotopes and Radiation Metrology Department (DRMR), IFIN Horia Hulubei, 023465 Măgurele, Romania; paulalbu@gmail.com (P.C.A.); razvan.ioan@nipne.ro (M.-R.I.); 4 Department of Bioengineering, University Sapientia of Miercurea-Ciuc, 500104 Miercurea Ciuc, Romania; tczszidonia@yahoo.com; 5 Department of Electronic Technology and Reliability, Faculty of Electronics, Telecommunications and Information Technology, University Politehnica of Bucharest, 061071 Bucharest, Romania

**Keywords:** thorium removal, thorium recovery, thorium recycling, thorium separation, thorium transport, thorium separation processes, thorium membrane separation, thorium membrane concentration, thorium determination

## Abstract

Although only a slightly radioactive element, thorium is considered extremely toxic because its various species, which reach the environment, can constitute an important problem for the health of the population. The present paper aims to expand the possibilities of using membrane processes in the removal, recovery and recycling of thorium from industrial residues reaching municipal waste-processing platforms. The paper includes a short introduction on the interest shown in this element, a weak radioactive metal, followed by highlighting some common (domestic) uses. In a distinct but concise section, the bio-medical impact of thorium is presented. The classic technologies for obtaining thorium are concentrated in a single schema, and the speciation of thorium is presented with an emphasis on the formation of hydroxo-complexes and complexes with common organic reagents. The determination of thorium is highlighted on the basis of its radioactivity, but especially through methods that call for extraction followed by an established electrochemical, spectral or chromatographic method. Membrane processes are presented based on the electrochemical potential difference, including barro-membrane processes, electrodialysis, liquid membranes and hybrid processes. A separate sub-chapter is devoted to proposals and recommendations for the use of membranes in order to achieve some progress in urban mining for the valorization of thorium.

## 1. Introduction

Thorium is a relatively exotic element, although it is known to have a significant natural abundance compared to lead [1]. With a component of the actinide series at position #90 and a weight of the gram atom equaling 232.03, it is unstable (radioactive) in all its isotopes except isotope ^232^Th [2]. The half-time of ^232^Th is so long that it is considered stable when joining uranium, which also occurs naturally [3]. The interest in thorium as a nuclear material should have resulted in an increased interest both on the part of researchers and on the part of energy producers [4]. However, the particularity of thorium is that it cannot sustain a chain reaction by itself, as is the case with uranium and plutonium [5], but fission can be produced under the influence of neutrons from an external source [6]. If the thorium atoms absorb a neutron, they turn into a heavier isotope, which then rapidly disintegrates into an isotope of the element protactinium and further into a fissioned uranium isotope under the incidence of bombardment with another neutron [7]. Because its disintegration line does not end with a material usable in the military industry, the interest in this nuclear fuel remains low [8].

Thus, the number of existing publications highlighted in Google Scholar [9] or SCOPUS [10], selected according to a specific algorithm [11] and using various keywords of scientific interest, is relatively moderate or even low (Table 1).

Recent publications are consistent and draw attention to the need to reconsider thorium as a nuclear material with a clear perspective [1,2,3,4,5] but also as an environmental polluter [6,7,8]. On the other hand, new materials usable in various analytical or technological separative techniques are also studied [12,13,14,15,16,17,18,19,20].

However, after analyzing Table 1, many would be discouraged to start research on aspects of recovery, recycling, or removal of thorium from various sources, although its common applications have determined its presence in urban waste in surprisingly high concentrations.

This last observation led to the initiation of this paper, whose aim is to warn both researchers and environmental officers regarding the danger of the uncontrolled spread of thorium as well as propose simpler solutions for removal, recovery and recycling, based on processes very close to “urban mining”.

The specific objectives of this work are to emphasize the existence of thorium in various materials used over time for common applications, the toxicity and bio-medical implications of thorium, the established technologies for obtaining thorium, the speciation of thorium in aqueous solutions, and the determination of thorium membrane processes with integration perspectives in thorium recovery or removal technologies and proposals regarding this aspect.

## 2. Applications of Thorium

**Figure 1:**
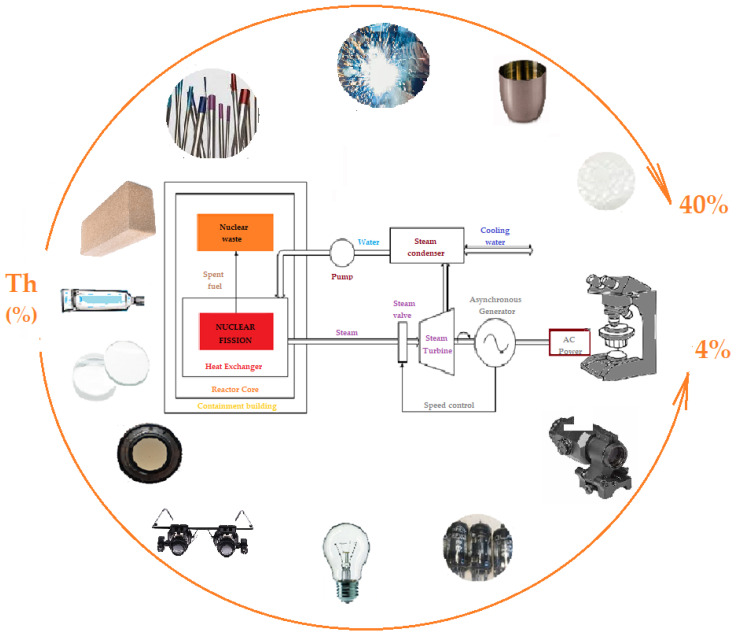
Domestic applications of thorium and thorium dioxide, along with the alleged use in generating energy in nuclear power plants (U–Th cycle).

Thorium, and especially thorium dioxide, has found relatively numerous applications for a radioactive element, even if this radioactivity is weak [21,22,23]. As various and unexpected, with many having been abandoned, the applications of thorium are so common (Figure 1) that they have become dangerous [21], especially since after the use of various materials and under the conditions of inattention in recycling or selective collection, thorium ends up in the environment [22].

In addition to its surprising use in toothpaste, in the dating of hominids, as a contrast agent in certain radiological examinations or as a filament in incandescent light bulbs, lamps, lanterns, and thorium mantles [23], it is also used for technical applications in which it is practically irreplaceable: crucibles for high temperatures, welding electrodes and alloys (aluminum, magnesium, steel), lamps for special electronic equipment, mantles in the metallurgical industry, industrial catalysts (ammonia, sulfuric acid, cracking hydrocarbons), the manufacture of thorium-mixed oxide tablets and uranium, oxygen detectors, and lenses for various optical and opto-electronic devices (having excellent wavelength dispersion and high refractive index) [24,25].

We can conclude that thorium, although radioactive, can be found in the aerospace industry, automobile industry, chemical and metallurgical industry, electrotechnical industry, electronics industry, dentistry (cements for dentistry, optical and surgical instruments, manufacturing), and in art objects (alloys, jewelry, sculptures, statues) [26,27], which leads to thorium being an environmental pollutant [28,29,30].

## 3. Toxicity and Bio-Medical Implications

Thorium is included on the list of carcinogenic substances [31], even though it decomposes through alfa decay [32], and the emitted alfa radiation cannot penetrate human skin [33].

The dangers associated with its radioactivity, due to the use of thorium in various technologies that capitalize on the high melting of thorium dioxide, lead to the following [34,35,36,37,38]:

- the amounts of thorium in the environment can be accidentally increased during processing;
- humans absorb thorium through food or drinking water (in areas adjacent to mining operations);
- the quantities in the air are very small (insignificant and generally neglected);
- amounts are high near hazardous waste storage or processing sites;
- amounts are high in industrial laboratories or mining laboratories that mill minerals containing thorium.

The medical effects, observed over time, of those who acquire thorium at work are as follows [39,40,41,42]:

- greater chance of developing lung disease;
- higher occurrence of lung and pancreatic cancer;
- changes in genetic material;
- higher instance of blood cancer;
- greater chance of developing liver diseases (when injecting thorium for X-rays);
- storage in bones (long-term exposure) can lead to the generation of bone cancer.

Being a heavy metal, the medical effects of thorium as well as the precautions for working with it must be considered [43,44].

At the same time, natural thorium is in secular equilibrium with its descendants, which makes it necessary to consider their radiotoxicity; for this reason, it is classified among the most dangerous radionuclides [45,46].

## 4. Classical Technology

Thorium is found in monazite (1 to 15%) in concentrations that allow it to be exploited on an industrial scale, through classical technologies [47]. At the same time, thorium appears in mining processes, especially those aimed at obtaining rare earths or uranium [48,49,50].

**Figure 2:**
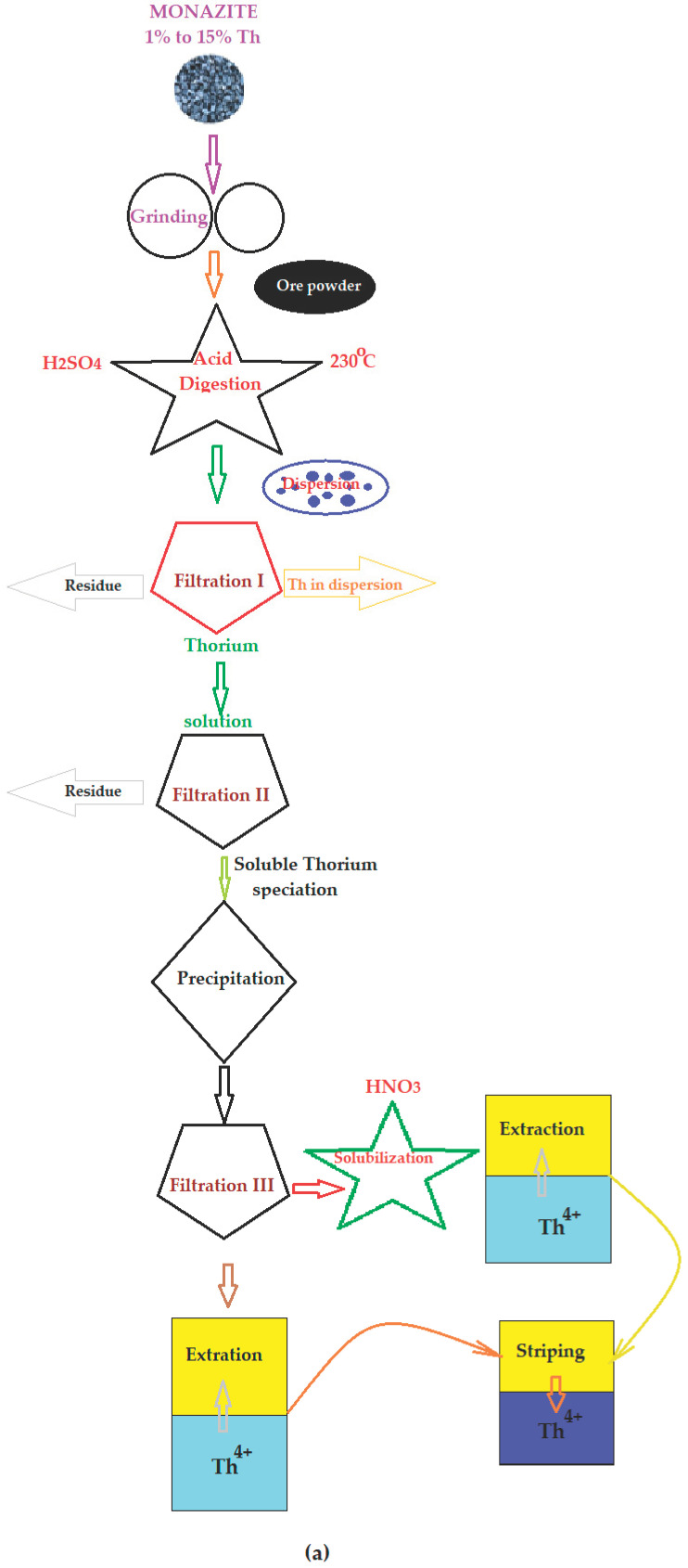

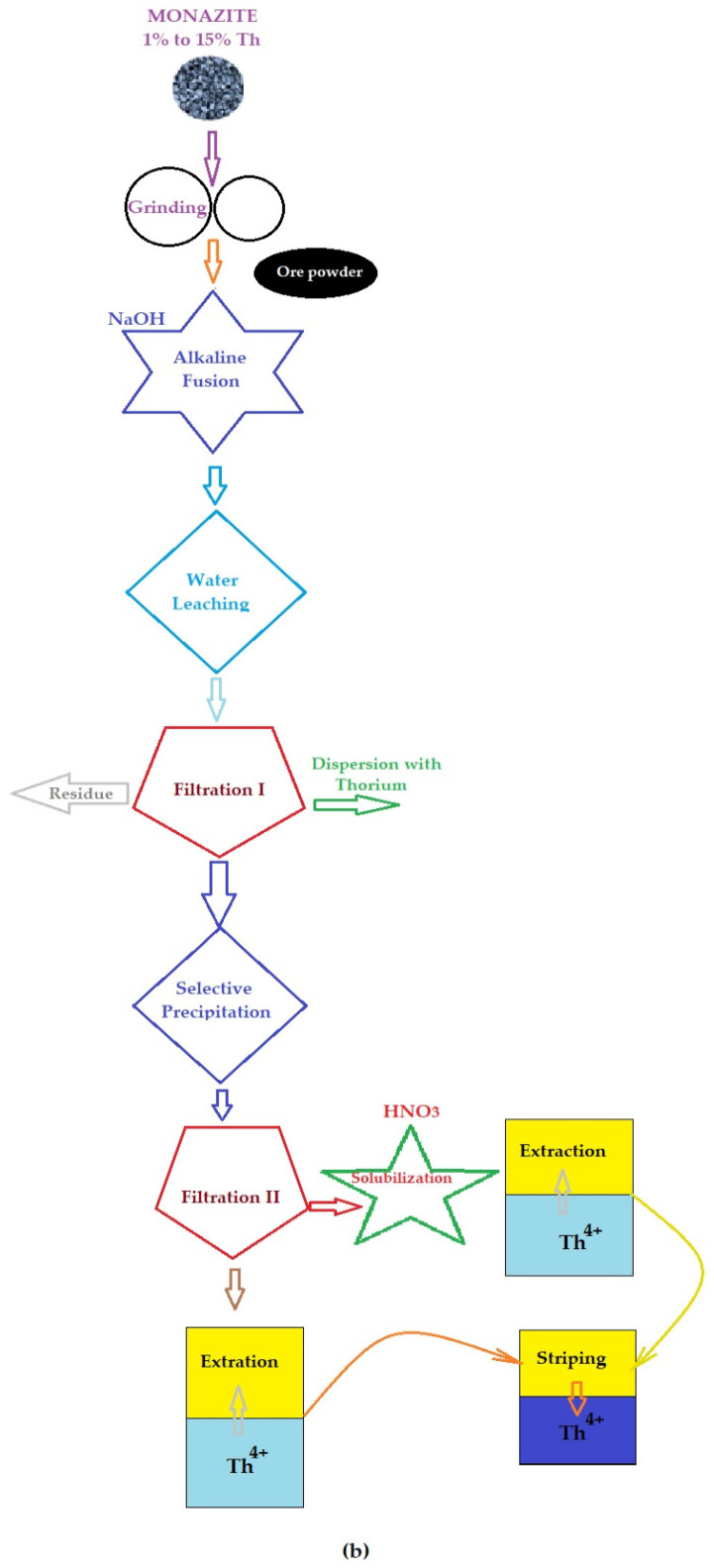
Simplified flowcharts for obtaining thorium from monazite: (**a**) acid digestion; (**b**) alkaline fusion.

The schemes in Figure 2 show the main operations that lead to obtaining thorium from monazite through acid (Figure 2a) or the basic attack (Figure 2b) of impurities; however, in principle, any mineral is considered as a source of thorium, with the series of technological operations being the same [51,52,53,54,55,56,57,58].

In practice, the mineral (source of thorium) is brought to a state of fine grinding in order to be attacked by sulfuric acid or a base (sodium hydroxide), so that the parts of the mineral not containing thorium pass into the solution, while others are removed by filtration. The filtrate containing thorium (colloidal) can be directly processed (when purification is not done in this technology) or precipitated, filtered and finally subjected to purification by extraction in an organic solvent (kerosene) and TBP as a complexant [59], or an organic solvent and an amine or a selective complexant and re-extraction [60].

If the source of thorium is a mineral containing rare earths or the residue obtained during the processing of various minerals in order to obtain rare earth elements (REEs), then the basic procedures used in the separation, concentration and purification of thorium are leaching [61,62,63], precipitation [64,65,66,67], solvent extraction [68,69] and ion exchange [70].

Obtaining thorium from pure compounds (halogens, halides) or alloys can be performed using physical (thermal) or chemical (reduction) processes [71,72].

For the current work, which involves obtaining thorium from industrial residues or by-products (waste), the diagrams in Figure 2 present, as narrow technological points, the filtration and extraction operations likely to be replaced to avoid environmental pollution [73,74].

## 5. Thorium Speciation

Thorium compounds are relatively few compared to other elements, even the less reactive ones [75,76]. Thus, thorium dioxide, halogens or a nitride are encountered, but the speciation of the thorium ion (Th^4+^) in aqueous solutions is of practical importance, as countless hydroxylated chemical species can be generated: [ThOH]^3+^, [Th(OH)_2_]^2+^, [Th(OH)_3_]^+^, [Th(OH)_4_], [Th(OH)_2_ (CO_3_)_2_]^2−^, [Th_2_(OH)_2_]^6+^ and [Th(H_2_O)_9_]^4+^ [77,78,79], hence the importance of the operational parameters (pH, ionic strength, temperature, contact ions in the aqueous solution [80,81,82,83]), which would be the object of the study of membrane processes.

**Figure 3:**
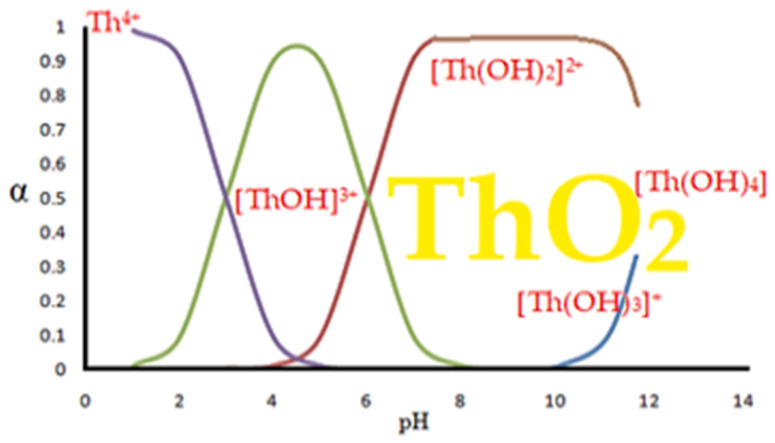
Hypothetical stability diagram of thorium hydroxo-complexes in aqueous medium.

When dissolving thorium nitrate (for example) in water, the mentioned hydroxyl species are formed, but also combinations that may include carbon dioxide (present in the environment). Considering the formation of only thorium hydroxides in aqueous solution, a series of chemical species are formed as a result of some equilibria with proton exchange, which is dependent on pH and is shown hypothetically in Figure 3. The degree of formation in solution of various chemical species can be determined exactly if the acidity constants of the chemical species and/or stability constants of the hydroxyl complexes are known [67].

The appearance of thorium dioxide is also related to the pH, ionic strength and temperature of the solution; however, as a solid phase, it depends essentially on the concentration of thorium obtained at a given moment in the phases of a technology, and more importantly, on the distribution of thorium in the environment [80,81,82,83].

**Figure 4:**
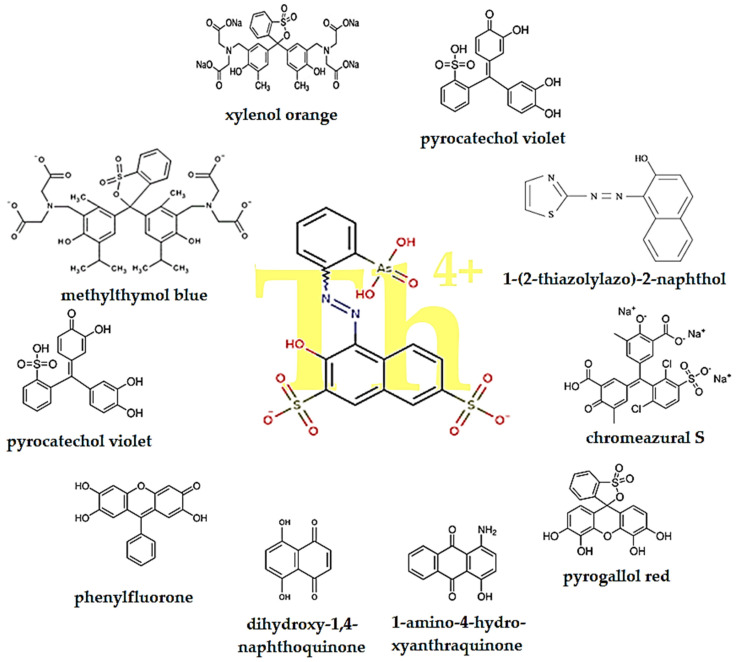
Common organic reagents involved in thorium ion complexation and/or extraction. The colored groups (in red) interact with the thorium ion (in yellow).

At the same time, if we consider that thorium is obtained in the source solution as Th^4+^ ion, then a wide series of organic complexants (Figure 4) can contribute to the formation of some speciations involved in the concentration and recovery, especially through extraction, of thorium [84,85,86,87,88,89,90,91,92,93,94,95,96,97,98,99,100,101,102].

The speciation of thorium in aqueous solution is important because the various hydroxo-hydroxyl species have different sizes, so membrane processes based on size separation can be chosen accordingly, moving from reverse osmosis to nanofiltration or even to ultrafiltration or colloidal filtration. Certainly, the aspects of chemical speciation of thorium in the presence of some inorganic complexants, but especially organic ones, are much more difficult to exploit, because the new chemical species have various hydrophobic-hydrophilic shells, depending on the considered ligand. These chemical species can be considered for separations with liquid or composite membranes that exhibit selective or even specific interactions with the ligands that incorporate the thorium ion.

## 6. Thorium Determination

Although a radioactive element, physical–chemical analysis based on specific reactions finds permanent use in various applications [86,87,88,89,90,91,92,93,94,95,96,97,98,99]. The main reagent, studied exhaustively and used with excellent results in various working conditions, is Thorin, 1-(2-Arsonophenylazo)-2-hydroxy-3,6-naphthalene-disulfonic acid sodium salt, 2-(2-Hydroxy-3,6-disulfo-1-naphthylazo)-benzene-arsenic acid sodium salt; Empirical Formula (Hill Notation): C_16_H_11_AsN_2_Na_2_O_10_S_2_ [87,88].

Otherwise, the radiation analyses for thorium are few, although they refer to the entire radiation register (α, β or γ) [103,104,105,106,107,108,109] (Table 2).

That is why, alongside the highly developed spectrophotometric methods [95], the studied reagents are today widely used for preconcentration [96], with a view to the permanent development of new methods, including electrochemical, optode, electrochemical sensor and coupled spectral methods [110,111,112,113,114,115,116,117,118,119,120,121,122,123,124,125,126,127,128,129,130,131,132,133,134].

**Table 2:** Analysis methods for thorium: characteristics and applications.

Analytical Methods	Samples and/or Applications	Characteristics	Refs.
Radiometric analysis (α, β or γ)	Determination of uranium, thorium, plutonium, americium and curium ultra-traces	Photon–electron rejecting α liquid scintillation	[103]
	Determination for levels of uranium and thorium in water along Oum Er-Rabia River	Alpha track detectors	[104]
	Thorium determination in intercomparison samples and in some Romanian building materials	Gamma ray spectrometry	[105]
	Thorium determination	Miscellaneous techniques	[106,107]
X-ray fluorescence spectrometry	Determination of thorium in natural water	Coupled with preconcentration method	[108]
	Trace element determination in thorium oxide	Total reflection X-ray fluorescence spectrometry	[109]
Inductively Coupled Plasma- (ICP-)	Analysis of rare earth elements, thorium and uranium in geochemical certified reference materials and soils	Mass spectrometry (ICP-MS)	[110]
	Determination of trace element concentrations and stable lead, uranium and thorium isotope ratios in in NORM and NORM-polluted sample leachates	Quadrupole-ICP-MS	[111]
	Chemical separation and determination of seventeen trace metals in thorium oxide matrix using a novel extractant—Cyanex-923	Atomic Emission Spectrometry (AES)	[112]
	Determination of Th and U	AES with MSF	[113]
	Determination of trace thorium in uranium dioxide	AES	[114]
	Determination of REE, U, Th, Ba and Zr in simulated hydrogeological leachates	AES after matrix solvent extraction	[115]
	Determination of thorium and light rare-earth elements in soil water and its high molecular mass organic fractions	MS and on-line-coupled size-exclusion chromatography	[116]
	Determination of trace thorium and uranium impurities in scandium with high matrix	Optical Emission Spectrometry (OES)	[117]
	Determination of thorium(IV), titanium(IV), iron(III), lead(II) and chromium(III) on 2-nitroso-1-naphthol-impregnated MCI GEL CHP20P resin	Preconcentration and MS	[118]
	Trace metal determination in uranium and thorium compounds without prior matrix separation	Electrothermal vaporization and AES	[119]
Atomic Absorption Spectrometry (AAS)	Thorium, zirconium and vanadium as chemical modifiers in the determination of arsenic	Electrothermal atomization	[120]
Cyclic Voltametric (CV)	Application in some nuclear material characterizations	Uranyl ion in sulfuric acid solutions	[121]
Chemically Modified Electrode (CME)	Determination of thorium by adsorptive type	Poly-complex system	[122]
Fluorogenic thorium sensors	Based on 2,6-pyridinedicarboxylic acid-substituted tetraphenylethenes	Induced emission characteristics	[123]
Selective optode	Design and evaluation of thorium (IV)	Membrane was prepared by incorporating 4-(*p*-nitrophenyl azo)–pyrocatechol	[124]
Micellar electrokinetic chromatographic	Ore and fish samples	Analysis of Th, U, Cu, Ni, Co and FE	[125]
Laser-induced breakdown spectrometry	Determination of trace constituents in thoria	Determination of thorium or uranyl ions	[126,127]
Electrochemical and spectro-electrochemical	Studies of bis(diketonate) thorium(IV) and uranium(IV) porphyrins	Complexes were synthesized using a hexa-aza porphyrin	[128]
Electrochemically modified detector	Elemental analysis of actinides	Graphite electrode with phthalocyanine	[129]
Selective extraction and trace determination of thorium	Synthesis of its application in water samples by spectrophotometry	UiO-66-OH zirconium MOF	[130]
Anodic polarization of thorium	Study of tungsten, cadmium and thorium electrodes	Electrochemical impedance spectroscopy	[131]
High-performance liquid chromatography	Studies on lanthanides, uranium and thorium	Amide-modified reversed phase supports	[132]
Ion exchange	Extraction of thorium on resin	Available extraction chromatographic resin	[133]
	Separation of actinium from proton-irradiated thorium metal	Extraction chromatography	[134]

The presented analytical methods offer, in addition to the information needed for the specific determinations of thorium in various matrices, possible ways of approaching separation through membrane techniques alternative to extraction, ion exchange, adsorption or chromatography.

## 7. Thorium Separation and/or Pre-Concentration

In analytical or technological research, the concentration of thorium has constituted a special problem, as it accompanies the rare earth elements (REEs), especially uranium [135,136].

The concentration process that is often followed and technologically supported is the pre-concentration of thorium [137,138,139].

Table 3 presents results that can form the basis of the development of urban thorium mining and that are focused on the concentration, pre-concentration, separation, extraction, ion exchange, sorption and bio-sorption of thorium or thorium–uranium from various samples and aqueous solutions [140,141,142,143,144,145,146,147,148,149,150,151,152,153,154,155,156,157,158,159,160,161,162,163,164,165,166,167,168,169,170].

The remarkable results in the development of organic ligands (Table 2), and especially of selective materials (Table 3), allow a confident approach to the recovery and recycling of thorium from electrical and electronic waste, but more generally (considering the slightly selective separation of waste) of residues that reach the integrated municipal storage and waste platforms (especially from construction).

## 8. Membrane and Membrane Processes

Membranes and membrane processes can be an attractive alternative for the separation of chemical species containing thorium from various sources, with reduced concentrations in this element. On the one hand, membranes can integrate into the classical technologies for obtaining thorium; on the other hand, the speculations that can be made between thorium and various complexants are compatible with membrane separations.

In order to highlight these aspects, this subchapter presents some characteristics of the main membrane processes.

Membranes and processes have evolved from laboratory-scale installations to industrial ones, having at the same time an increased economic and commercial importance [170]. Membrane processes have not only replaced some of the conventional separation processes but also have produced remarkable results in areas where conventional techniques are exhausted or very expensive [171]. Among the problems that have determined the exponential development of membrane processes are those of environmental protection, since technologies based on membranes and membrane separation techniques are recognized as ecological technologies [172].

### 8.1. Introduction to Membranes and Membrane Processes

**Figure 5:**
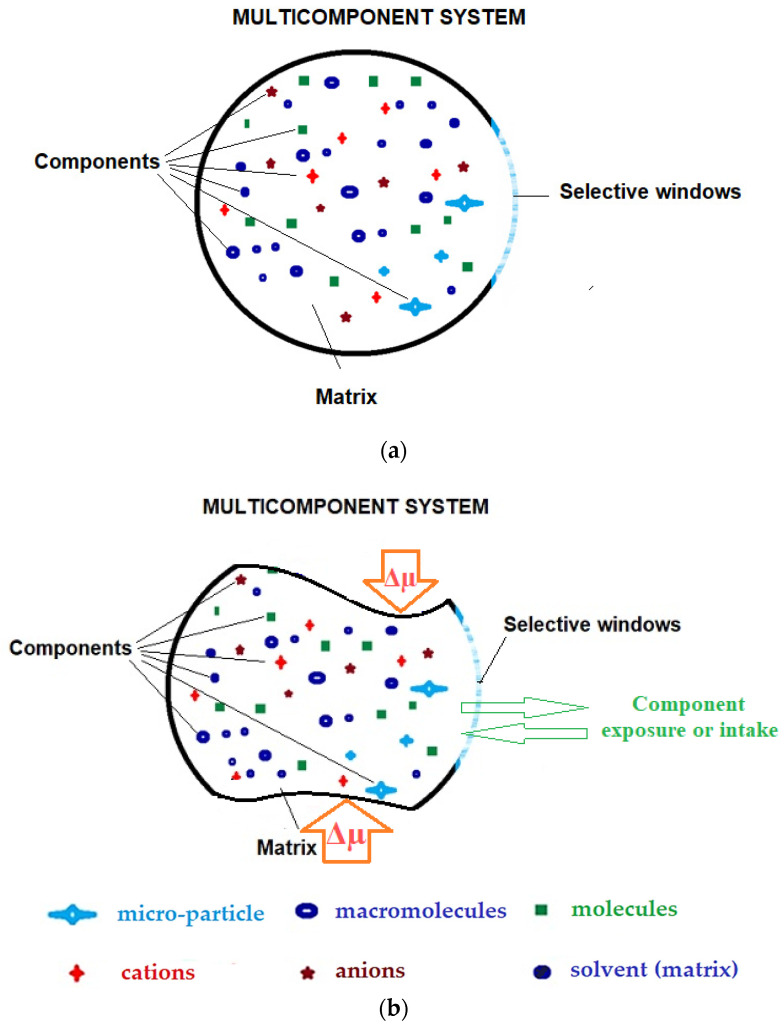
Multicomponent system bordered by a selective window, including ions, small molecules, macromolecules, nanoparticles, microparticles, microorganisms and viruses as suspended particles: (**a**) system in equilibrium; (**b**) system subject to an electrochemical potential difference (Δµ). The meaning of shapes and symbols in Figure 5 is as follows.

If we focus on membrane processes, it can be stated that the membrane is a window of a multi-component system (Figure 5), with selective permeability for chemical species of the system [173]. This membrane allows the separation of the considered system, consisting of a continuous phase (solvent) in which ionic chemical species, molecules and macromolecules are dissolved. At the same time, molecular aggregates and dispersed particles can be separated into components by classical or membrane processes [174]. In order for the separation process to occur, the system must be subjected to an electrochemical potential difference or driving force (Δµ) [175].

The most important driving forces on membrane processes are as follows [176]:

- P = transmembrane pressure difference;
- Δc = concentration difference between the two compartments separated by a membrane;
- ΔE = potential difference.

It should be emphasized that in the last decade membrane processes involving potential gradient, thermal, magnetic, and interfacial tension, and volatility have undergone significant development [177].

In this subchapter, we will briefly present the essential aspects of the processes involving pressure or concentration gradient (liquid membranes).

### 8.2. Barro Membrane Processes

**Figure 6:**
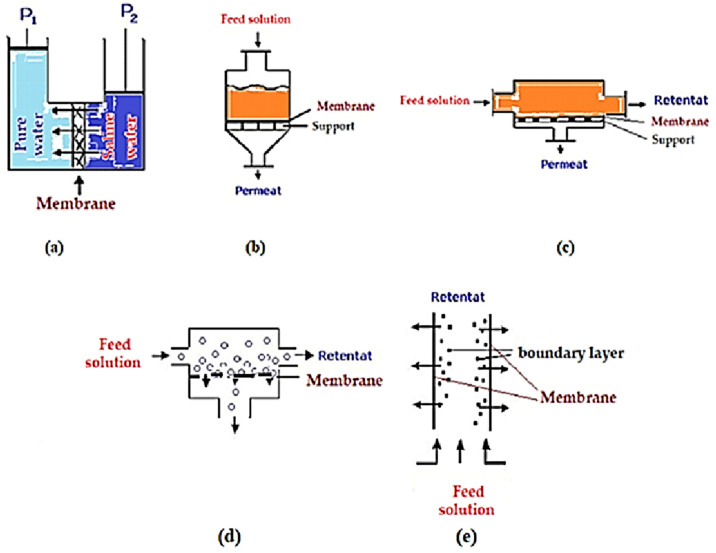
Membrane separation processes under pressure difference: (**a**) obtaining drinking water through reverse osmosis; (**b**) piston type (dead-end filtration); (**c**) tangential flow; (**d**) tangential flow through large sections; (**e**) flow through tubes.

In membrane processes, the pressure difference (Δp) constitutes a technically and economically accessible driving force, leading to many applications, including microfiltration, ultrafiltration, nanofiltration and reverse osmosis (hyperfiltration) [178]. The first and most developed application was the obtaining of drinking water from sea water (Figure 6a), when it was found that, by applying a pressure higher than the osmotic pressure of sea water, most of the solvent passes (96–99%) through a semi-permeable membrane [179]. While these processes have applications on an industrial scale, their introduction in a certain technology presents a flow optimization problem (Figure 6b,c) [178,179,180], which depends on the load in the chemical species to be removed from the solvent that constitutes the feed [181]. There is the option of operating using dead-end filtration of a cross-flow filtration system [182]. The design of filtration devices may differ; chemical equipment manufacturers compete to create prototypes with increasingly high performance by improving the flow on the membrane (Figure 6d,e) [183,184]. In filtration processes, regardless of preventative efforts, the membrane becomes dirty or clogs, or concentration polarization (solute accumulation) occurs on the layer adjacent to the membrane; thus, process engineering is complemented by the introduction of ultrasonic cleaning devices into the technology, cavitation, magnetic stirring, or pulsatile flow vibrations [185].

**Figure 7:**
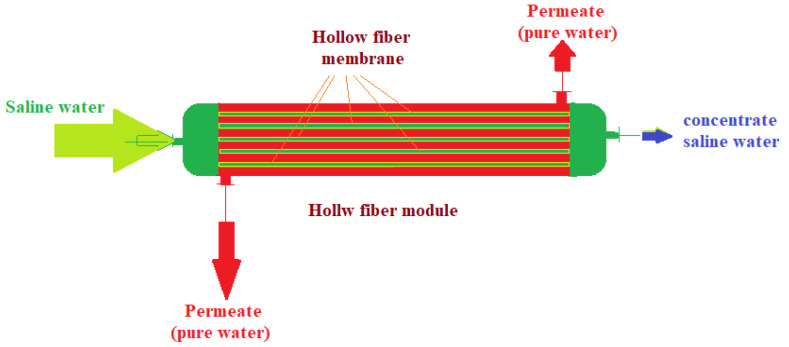
Advanced hollow-fiber filtration module.

However, in essence, the feeding can be done through large cylindrical, tubular, spiraled or capillary (hollow fiber) spaces, in which, along with the flow through and/or on the membrane and avoiding fouling (contamination, soiling), the aim is to increase the area of the contact surface of the membrane with the dispersed system of feeding (Figure 7) [186]. Of course, the operation can be done by introducing the feeding solution, as in Figure 7, but most often, with the feed solution being dirtier, it is inserted between tubes or fibers for a possible physical cleaning [187,188,189].

A homogenous system can be separated by aggregation (segregation), so that instead of a high-pressure process (Table 4), a lower-pressure one is used [186,187].

The first processes of this kind were promoted by Schamehorn, the ultrafiltration of micellar systems (MUF), which consisted of transforming a solution into an ultra-micro-dispersed system by adding suitable surfactants, followed by ultrafiltration [190,191,192].

The condition for using micellar ultrafiltration is that the micelles contain the organic compound, which means an impurity of the concentrate [193].

**Figure 8:**
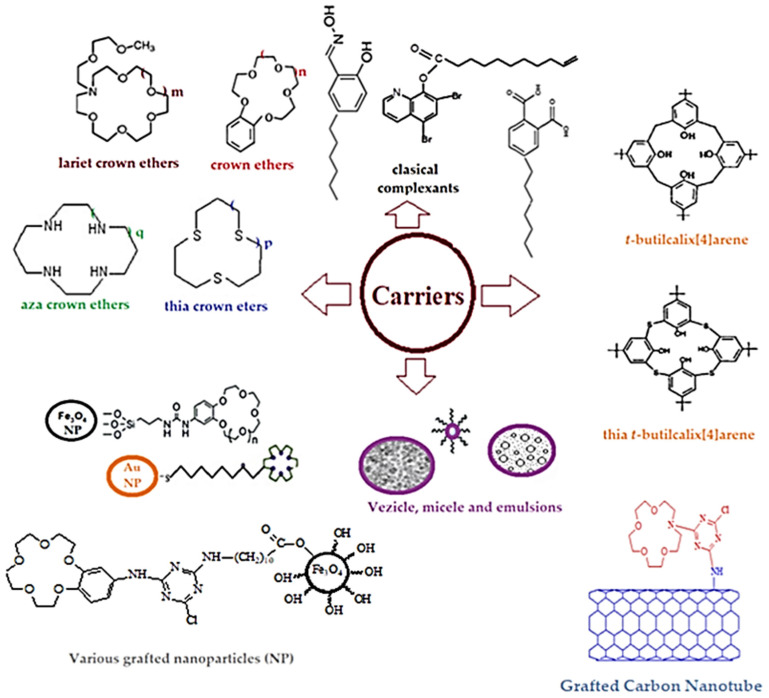
Common types of carriers: macrocyclic compounds, modified classical complexant agents and nano-species [194].

The variants of ultrafiltration and nanofiltration have undergone significant development due to nano-species and nanomaterials (nanoparticles, nanotubes, nanofibers, proteins, soluble polymers, polyelectrolytes, micelles and vesicles) also being used as carriers (Figure 8) in processes in liquid membranes [194].

The concentration polarization and the diffusion effects related to the sizes of solutes with low molecular masses can influence the working conditions of nano- and ultrafiltration, with the number of additives required being determined experimentally [195].

### 8.3. Electro-Membrane Processes

**Figure 9:**
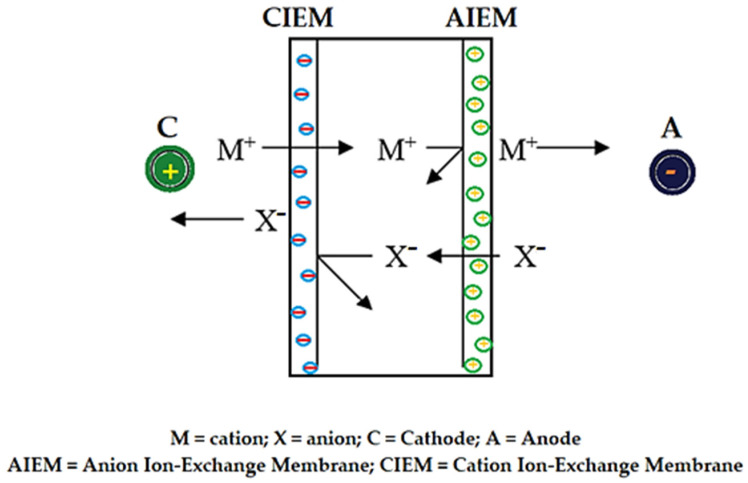
Scheme of an electrolysis cell for the concentration of a salt by electrodialysis with two ion exchange membranes.

Electrodialysis is the most widespread separation process, carried out under an electric potential gradient, which involves ion exchange membranes [196]. In electrodialysis, the extraction, reconcentration and substitution operations are carried out without direct intervention of the electrodes [197]. They are placed at the end of the electrodialysis cells in order to maintain the electric potential difference between the compartments separated by the membranes (Figure 9) [197,198,199].

If we associate an anion exchange membrane with a cathode, it is possible to eliminate an electrolyte, whose cation can be deposited by electrochemical reaction on the cathode [200]. The electrolyte extracted from the diluted circuit by electrodialysis will be recovered in the concentrated circuit according to the principle in Figure 9. This electrolyte will not only be recovered but can be reconcentrated. Recovery and reconcentration are possible because the ions cannot migrate over their compartment, the M cation being retained by the anion exchange membrane, and the X anion by the cation exchange membrane [201,202].

Conducting electrodialysis requires ways to interpose electrodes, aqueous phases to be processed, and membranes, so that the operation can lead at the same time to solute concentrations or to the recovery of deionized water [203,204,205].

### 8.4. Membrane Processes Carried out under a Concentration Gradient (Liquid Membrane)

Although the concentration gradient is also found in processes with solid membranes (osmosis, dialysis, forward osmosis), this paper addresses processes with liquid membranes that have a high chance of developing applications in the valorization of thorium [206].

Separation systems with a liquid membrane (LM) or bulk liquid membrane (BLM) are formed by two homogenous liquid phases, immiscible with the membrane, called the source phase (SP) and the receiving phase (RP). The separation of the two liquid membranes is achieved with a third liquid, the membrane (M), which acts as a semi-permeable barrier between the two liquid phases [207,208,209,210,211,212,213,214,215,216,217,218,219,220,221,222,223,224,225,226,227,228,229,230,231].

**Figure 10:**
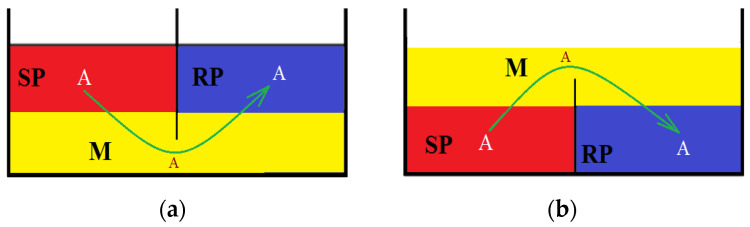
Schematic presentation of membrane systems with an organic solvent: denser (**a**) or less dense (**b**) than aqueous phases. Legend: M = membranes; SP = source phase; RP = receiving phase; A = chemical species of interest for separation [214].

An established graphic but also practical conception of liquid membranes (Figure 10) takes into account the density of the membrane, which is generally an organic solvent or a multicomponent system in which the continuous phase is the organic solvent [208].

The density of the membrane phase becomes unimportant if the membrane solvent is immobilized in or on a support [209], thus obtaining supported liquid membranes (SLMs). An interesting variant, but not yet sufficiently evaluated in separation processes, is the liquid membrane based on magnetic liquid (ferrofluid) [210], which also has no restrictions on the density of the organic solvent but involves special aspects in terms of stability and the transfer of table [211].

If we focus on BLMs, the technical problems to be solved are as follows: the large volume of solvent used (V), the small mass transfer area (σ), the unit ratio between the volume of the source phase, the volume of the receiving phase (r) and the volume of the membrane organic solvent (OS or M) and, therefore, implicitly, the long operating time (t) [212].

**Figure 11:**
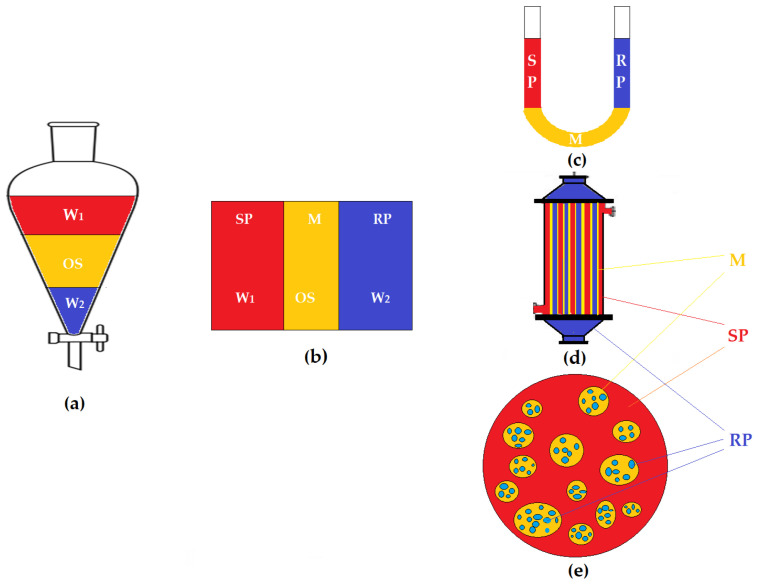
Schematic presentation of extraction and membrane systems with organic solvent: (**a**) water 1 (W1)–organic solvent (OS)–water extraction (W2); (**b**) liquid membranes (LMs); (**c**) bulk liquid membranes (BLMs); (**d**) supported liquid membranes (SLMs); (**e**) emulsion liquid membranes (ELMs). Legend: M = Membrane; SP = Source Phase; RP = Receiving Phase [214,216].

In order to improve the performance, hollow-fiber supported membranes (HFLMs) and emulsion membranes (ELMs) have been greatly developed (Figure 11) [213].

**Figure 12:**
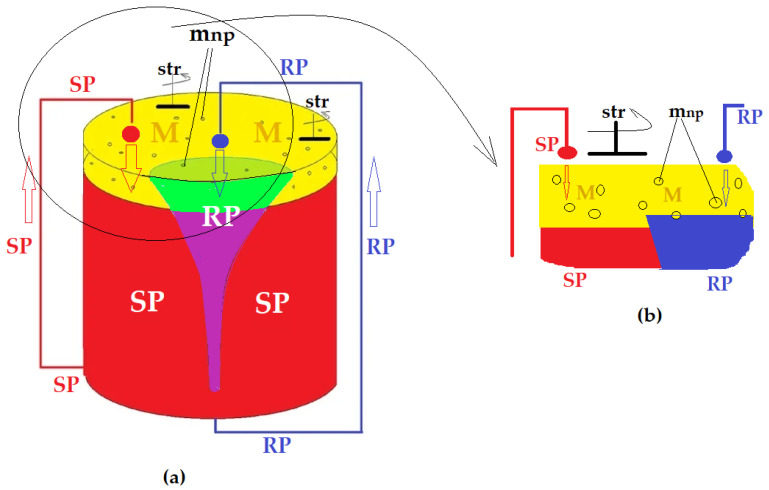
Schematic presentation of the permeation module with dispersed phases: (**a**) front view; (**b**) cross-section detail. Legend: SP—source phase; RP—receiving phase; M—organic solvent membrane; m_np_—magnetic nanoparticles; str—stirrer with magnetic rods [216,217].

Recently, a BLM system with dispersed phases was studied, in which the aqueous phases of the separation system dispersed in/through the membranes. The membrane is a nanodispersed system of magnetic nanoparticles that have the role of ensuring both convection and transport for ionic chemical species in membranes based on saturated alcohols C_6_–C_12_ [214,215]. The most recent design is shown in Figure 12, but other variants using chemical nano-species are also used [216].

The BLM system with dispersed phases (based on Figure 8 carriers, for example) is close to the performance of liquid membranes on hollow-fiber supports or emulsion-type liquid membranes but has several limitations that restrict its applicability, including the stability of the membrane nanodispersion, control of the size of droplets in recirculating aqueous phases and losses of membrane material (solvent or nanoparticles) [217].

### 8.5. Transport in Liquid Membranes

**Figure 13:**
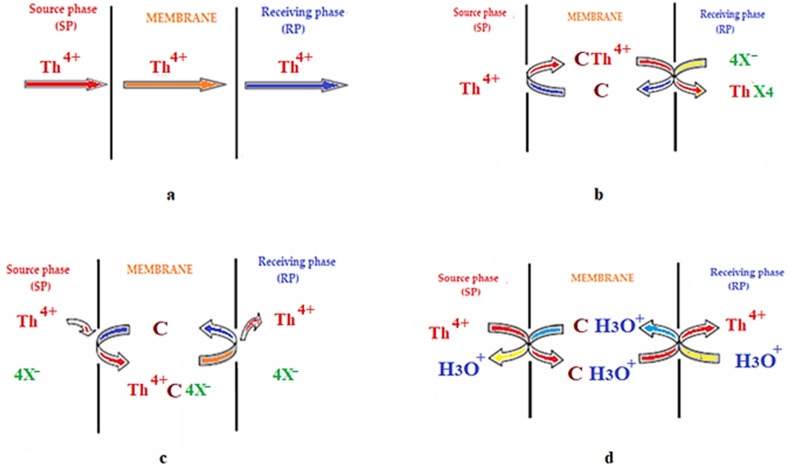
Schematic presentation of the transport mechanism by liquid membranes (C—carrier, X—anion complexant): (**a**) physical “simple” shipping; (**b**) transport with carrier; (**c**) coupled transport; (**d**) counter-transport.

The method of achieving the concentration gradient and the nature of the species dissolved in the phases of the membrane system have led to various types of transport through liquid membranes [218,219,220]. Mainly, however, they can be narrowed down to those specified in Figure 13.

#### 8.5.1. Physical “Simple” Shipping

The simple diffusion type of transport through the solution is usually followed by the permeation of the solute through the liquid membrane due to the concentration gradient (Figure 13a). In this case, the transport of the component from the source phase through the membrane phase occurs with a higher solubility or diffusivity of the solute in the membrane phase. In this type of transport, the mass transfer rate is low and depends on the solubility of the solute in the organic phase, as well as the solubility of the solute in the source and receiver phases [221,222].

#### 8.5.2. Facilitated Transport or Carrier-Mediated Transport

In carrier-mediated transport, a carrier is added to the membrane phase in order to increase the mass transfer rate or separation efficiency of the liquid membrane. It is also known as facilitated transport or transport mediated by a transporter [223]. In this case, the solute dissolved in the source phase, at the source phase–LM interface, reacts chemically with a transporter dissolved in the liquid membrane to form a complex. This complex reacts inversely at the LM–receiving phase interface, releasing the partitioned solute in the receiving phase (Figure 13b). In recent years, this type of transport mediated by a transporter has been intensively developed for the selective transport of cations, anions and neutral species through liquid membranes [224].

#### 8.5.3. Coupled Co- or Counter-Transport

In this type of transport, the transport speed of a certain ion is dependent on the concentration of another ion. In the case of coupled co-transport, the metal ion is transferred together with a counter-anion, with the two species’ transport taking place in the same direction. In the coupled counter-transport type, the simultaneous transport of another ion from the receptor phase to the source phase takes place; thus, the transport of the two species takes place in opposite directions [225,226,227]. Figure 13c,d shows the types of co- and counter-coupled transport of a metal ion.

### 8.6. Hybrid Membrane Processes

The most common form of treatment of effluents containing heavy metal ions involves the precipitation of metals as a hydroxide, base salt or sulfur. Precipitation is often followed by an additional treatment, such as sedimentation or filtration processes [228,229,230].

The technique of liquid membranes also presents a huge potential for the application of the removal and valorization of heavy metals, especially for the purpose of environmental protection [221,222,223].

Currently, the most important commercial application of liquid membrane technologies is the treatment of wastewater and waste [212,224,225,226].

However, the use of liquid membranes has encountered many obstacles, mainly related to the use of solvents with high toxicity [227,228]; both the reduction of the amount of the membrane solvent required and their replacement with green solvents or nanodispersions have created better opportunities for this process [229,230].

The idea of using nanosystems has led to the development of hybrid processes, which basically follow the mechanism of liquid membranes, but the process design is more advanced [211,231].

## 9. Problems in Application and Achievement as Well as Development Perspectives of Urban Thorium Mining

The analysis of the processes in which the minerals or waste containing thorium are processed shows that the classic technologies have material losses in the environment, which could be reduced with or through membrane techniques [232,233,234,235,236,237,238,239,240,241,242,243,244,245,246,247,248,249,250,251,252,253,254,255]. Thus, in the classical thorium recovery technologies, some disadvantages [1,13,14,15,16,17,18,19,20,47,48,49,50,51,52,53,54,55,56,57,58,59,60,61,62,63,64,65,66] of the operations are highlighted (Table 5), which require improvements, especially from the perspective of the loss of thorium in the environment.

The problem of thorium separation, concentration and recycling can be approached by analyzing some of the contributions that offer both priority research directions and viable technical solutions (Table 6) [232,233,234,235,236,237,238,239,240,241,242,243,244,245,246,247,248,249,250,251,252,253,254,255].

Recent studies on the separation, concentration, removal or recovery of thorium from aqueous solutions, including by membrane techniques [256,257,258,259,260,261,262,263,264,265,266,267,268,269,270,271] (Table 7), have led to promising results, reinforcing the idea that membrane or hybrid processes can contribute to the imaging of the technological recycling of thorium from various residues, especially industrial, on municipal waste processing platforms.

The various compounds [272,273,274,275,276,277], technologies and processes [272,273,274,275,276,277,278,279,280,281,282,283,284,285] proposed recently, but also some previously used [286,287,288,289,290,291,292,293], can contribute to the construction of a scheme for recuperative separation of thorium on an integrated municipal platform for processing, mainly the waste of electrical devices (lamps, tubes and mantles) and electronics, but also those from the construction industry (welding electrodes, metallic materials and alloys).

**Figure 14:**
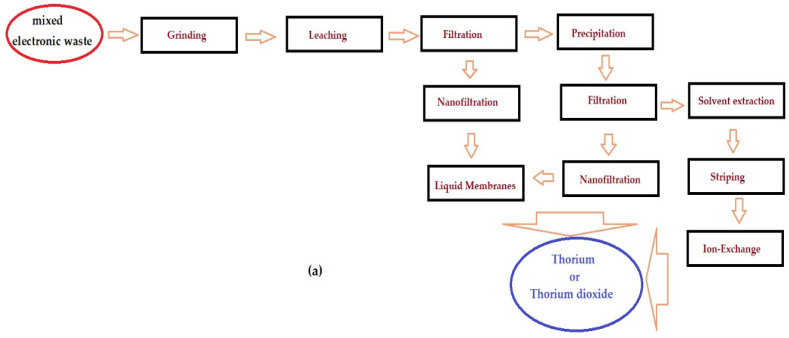

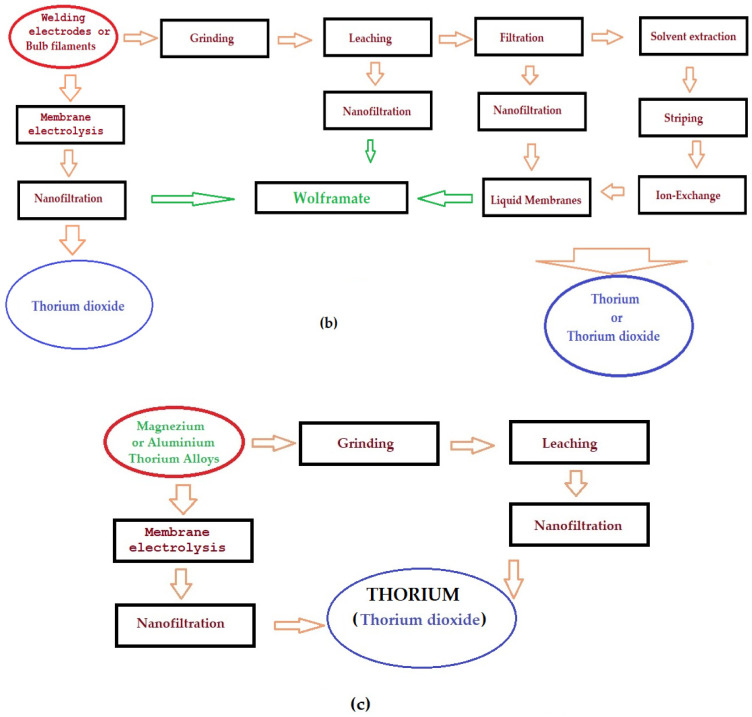
Scheme of proposals for the separation, recovery and recycling of thorium from waste of municipal waste management platforms: (**a**) valorization of thorium from unsorted waste; (**b**) recovery of thorium from electrodes and light bulb filaments; (**c**) valorization of thorium from magnesium or aluminum alloys.

In the diagrams in Figure 14, several proposals for technical solutions for urban thorium mining are presented, starting from the raw material: waste that ends up at integrated municipal waste management platforms.

Thus, in a first case (Figure 14a), it is assumed that the waste (assumed to be electrical and electronic waste or metal waste from metal construction materials) contains thorium and is totally unselected. This option would require the use of the classic scheme for the separation of thorium from poor sources, including the following operations: leaching, filtration (sedimentation), precipitation, filtration, solubilization at Th^4+^, extraction, re-extraction and ion exchange. In this operating scheme, membrane processes that can be integrated to increase the performance of the process are nanofiltration and/or liquid membranes.

A second case may be a raw material containing thorium alloyed with tungsten (filaments of incandescent lamps or other lighting fixtures, welding electrodes or building material alloys). Figure 14b shows the main operations, which consist of leaching, filtration, precipitation, filtration, nanofiltration, solubilization at Th^4+^, extraction and stripping or membrane electrolysis and nanofiltration.

The third possible case would be a raw material consisting of various wastes from aluminum and magnesium alloys (Figure 14c). Such a waste content can be processed for thorium recovery by membrane electrolysis or acid attack, followed by filtration and nanofiltration.

The proposed operation schemes are highly dependent on the quality of the waste selection that reaches the integrated municipal waste for management and processing platforms.

Certainly, some selection criteria for residues containing thorium can be taken into account in order to approach a treatment scheme as close as possible to the technological flows dedicated to obtaining this element.

Thus, we can consider that the entire deposit where thorium components were found, with a concentration above 1%, can be treated according to the classic acidic or basic digestion schemes.

When the waste deposit receives metal waste (residues of welding electrodes, metal alloys of thorium with aluminum or magnesium), a nitric acid digestion scheme followed by extraction and/or ion exchange will be required.

## 10. Conclusions

Although a radioactive element and a promising raw material for nuclear power generation, thorium, being a fairly abundant metal (similar to lead), has surprising domestic uses: toothpaste, dental cement, crucibles for high-temperature work, filaments for incandescent bulbs, welding electrodes, aluminum or magnesium alloys, jewelry, sculptures, coats and goggles, devices working at high temperatures and lamps for electronic devices.

Current regulations consider thorium to be a carcinogenic element, and its bio-toxicity and impact on human health (affects internal organs and blood) require the recovery and recycling of thorium, especially in the case of waste from municipal management platforms.

Classical thorium recovery processes require acid or base attack on thorium-containing feedstock, filtration, re-solubilization, extraction and ion exchange.

A variety of complexants and transporters have been used for the separation and preconcentration of thorium (especially for its analysis), which leads to membrane applications (nanofiltration, colloidal ultrafiltration, liquid membranes, emulsion membranes) for thorium utilization.

Membrane processes can intervene throughout the thorium recovery and recycling stream, increasing the efficiency of the process and avoiding losses to the environment.

The proposed processing schemes for various wastes containing thorium highlight the possibility of removal, recovery and valorization of thorium, suggesting possible urban mining of this element.

## Figures and Tables

**Table 1:** The number of publications highlighted in Google Scholar on various keywords related to thorium.

Keywords *	Scholar Google Publication Number in Different Periods			SCOPUS Publication Number
	Any Time	2014–2023	2021–2023	1995–2023
Thorium separation	162,000	82,000	12,900	2186
Thorium concentration	199,000	12,200	6200	7888
Thorium recovery	79,000	17,900	9200	896
Thorium removal	62,000	17,500	13,800	132
Membrane thorium separation	21,900	10,600	3730	458
Membrane thorium concentration	27,600	19,000	4610	141
Membrane thorium recovery	18,000	8600	3850	27
Membrane thorium removal	21,600	12,000	4500	5
“Thorium separation”	883	244	79	25
“Thorium recovery”	611	204	87	34
“Thorium recycling”	50	18	4	2
“Thorium membrane”	7	2	–	2

* accessed on 24 June 24 2023.

**Table 3:** Concentration and separation of thorium using various techniques and selective materials.

Processes/Methods/ Techniques	Materials	Characteristics	Refs.
Thorium removal	Different adsorbents	Activated carbons and zeolites (natural and synthetic)	[140]
Removal of thorium (IV) from aqueous Solutions	Modification of clinoptilolite as a robust adsorbent	Highly efficient thorium removal material	[141]
Preconcentration of uranium in natural water samples	New polymer with imprinted ions	Determination by digital imaging	[142]
Adsorption of trace thorium (IV) from aqueous solution	Mono-modified β-cyclodextrin polyrotaxane	Using response surface methodology (RSM)	[143]
Preconcentration and separation of actinides	Novel malonamide-grafted polystyrene-divinyl benzene resin	For hexavalent and tetravalent actinides such as U (VI), Th (IV) and Pu(IV)	[144]
Comparative adsorption	Mesoporous Al_2_O_3_	Selectivity of Th (IV) compared U (VI), La (III), Ce (III), Sm (III) and Gd (III)	[145]
Extraction and precipitation agents	α-aminophosphonates, -phosphinates, and -phosphine oxides	For rare earth metals, thorium, and uranium	[146]
Removal of polyvalent metal ions	Polyurea-crosslinked alginate aerogels	Eu (III) and Th (IV) from aqueous solutions	[147]
Method for separating thorium	Patented Chinese method	Separating cerium-fluoride and thorium	[148]
Extraction and recovery of cerium (IV) and thorium (IV)	α-aminophosphonate extractant	Extraction and recovery of Ce (IV) and Th (IV) from sulphate medium	[149]
Selective extraction and separation	Sulfate medium using Di(2-ethylhexyl)-N-heptylaminomethylphosphonate	Ce (IV) and Th (IV) from RE (III)	[150]
	α-aminophosphonate extractant	Ce (IV) from thorium and trivalent rare earths	[151]
	α-aminophosphonic acid HEHAPP	Heavy rare earths from chloride medium	[152]
	α-aminophosphonic acid extractant HEHAMP	Rare earths from chloride media	[153]
Study of thorium adsorption	PAN/zeolite composite adsorbent	Adsorption model	[154]
	Tulul Al-Shabba Zeolitic Tuff, Jordan	Adsorption of Th (IV) and U (VI)	[155]
	Sodium clinoptilolite	Removal of Th from aqueous solutions	[156]
	Modification of zeolite	Using tandem acid-base treatments	[157]
Selective cloud point extraction of thorium (IV)	Tetraazonium-based ionic liquid	Thorium extraction isotherm	[158]
Removal of thorium (IV) from aqueous solutions	Deoiled karanja seed cake	Optimization using Taguchi method	[159]
Retention of uranyl and thorium ions from radioactive solution	Peat moss	Retention of uranyl and Th ions from radioactive solution	[160]
Photocatalysis and adsorption	Photo-responsive metal-organic frameworks (MOFs)	Design strategies and emerging applications	[161]
Electrochemical and electrolytic separation	Th (IV) and Ce (III) in ThF_4_^−^CeF_3_-LiCl-KCl quaternary melt	Separation of Th (IV) and Ce (III)	[162]
Selective removal	Hybrid mesoporous adsorbent as benzenesulfonamide-derivative@ZrO2	Thorium ions from aqueous solutions	[163]
Extraction	Sodium diethyldithiocarbamate/polyvinyl chloride	Rare earth group separation from lamprophyre dyke leachate	[164]
Fluorescent sensors	Metal-organic framework (MOF)	Hazardous material detection	[165]
Zeolite adsorption	Separation of radionuclides	From a REE-containing solution	[166]
Equilibrium study	Acidic (chelating) and organophosphorus ligands	Equilibrium constants of mixed complexes of REE	[167]
Molecule for solvent extraction of metals	Thenoyltrifluoroacetone	Thorium extraction	[168]
Chemical adsorption	8-Hydroxyquinoline immobilized bentonite	Removal of U and Th from their aqueous solutions	[169]

**Table 4:** Characteristics of pressure gradient processes.

Type of Membrane Process	Pore Diameter (nm)	Pressure (Bar)	Obtained Water Content
Reverse osmosis	<0.6	25–60	Pure water (poorly ionized)
Nanofiltration	0.5–10	6–30	Pure water (traces of molecular substances)
Ultrafiltration	7–200	4–15	Pure water, molecular substances and macromolecules
Microfiltration	150–5000	0.1–2.5	Pure water, molecular substances and colloids

**Table 5:** Possible losses of thorium in the environment and remedial possibilities.

Technological Operation	Losses of Thorium or of Thorium-Contaminated Materials	Means of Remediation or Reduction of Losses
Crushing, grinding	Dust removal Mill shutdown losses Losses when cleaning the machine	Microfilter installation Micro- and ultrafiltration of colloidal washing solutions
Solubilization or leaching	Incomplete solubilization with the chosen reagent Complete solubilization Insufficient concentration of thorium	Solubilization with a complementary reagent Selective reprecipitation and solubilization Concentration by precipitation and microfiltration
Filtration	Thorium retention in the precipitate Reduced concentration of thorium in the filtrate	Washing with solubilizing reagents Reprecipitation and micro- or ultrafiltration
Precipitation	Incomplete precipitation Precipitation of nanometric particles	Nanofiltration or reverse osmosis of the filtrate Colloidal ultrafiltration or nanofiltration
Extraction	Solvent losses Incomplete extraction	Solvent recovery Use of selective extractants
Ion exchange	Blockage of thorium in the ion exchanger (elution inefficiency) Incomplete retention	Change eluent Recovery of ion exchangers for destruction (burning)

**Table 6:** Aspects regarding the use of membrane techniques and membrane materials, with possible implications regarding thorium separation.

Membrane Techniques	Materials and Applications	Characteristics	Refs.
Waste Treatment	Liquid radioactive waste treatment		[232]
Liquid Filtration	Membrane surface patterning as a fouling mitigation	Strategy for Processes	[233]
Ionic Liquid	Gas separation membranes		[234]
	Proton exchange membrane in fuel cells		[235]
	Chitosan-based polymers as proton exchange	Roles of Chitosan-Supported Polymers	[236]
	Based electrolytes for energy storage devices		[237]
	Toxicity to living organisms		[238]
Polymer Inclusion Membranes (PIMs)	Sequential determination of Copper (II) and Zinc (II) in natural waters and soil leachates	Chelating Resin	[239]
	Application in the separation of non-ferrous metal ions	Membranes (PIMs) Doped with Alkylimidazole	[240]
	Poly(vinylidene-fluoride-co-hexafluoropropylene) extraction from sulfate solutions	Containing Aliquat^®^ 336 and Dibutyl Phthalate	[241]
Bulk Hybrid Liquid Membranes	Operational limits	Based on Dispersion Systems	[217,242]
	Thorium transport: modeling and experimental validation	Continuous Bulk Liquid Membrane Technique	[243]
Membrane Fabrication	Sustainable membrane development	Polymers and Solvents Used	[244]
Light-Responsive Polymer Membranes	Miscellaneous application	Report Recent Progress In The Research Field	[245]
Adsorptive Membranes and Materials	Modern computer applications	Model for Rare Earth Element Ions	[246]
Nanofiltration	Effect of the adsorption of multicharge cations on the selectivity	NF and Adsorption	[247]
	Extraction of uranium and thorium from aqueous solutions	NF and Extraction	[248]
	Removal of fluoride	By Nature, Diatomite From High-Fluorine Water	[249]
	Removal of radioactive contamination of groundwater, special aspects and advantages	Including RO	[250]
	U from seawater by nanofiltration	Selective Concentration	[251]
Glutathione-Based Magnetic Nanocomposite	Sequestration and recovery of Th ions	Using Recyclable, Low-Cost Materials	[252]
Zeolite Hybrid Adsorbent	Case study of thorium (IV)	Evaluation of Sodium Alginate/Polyvinyl Alcohol/Polyethylene Oxide/ZSM5 Zeolite Hybrid Adsorbent	[253]
Functionalized Maleic-Based Polymer	Thorium (IV) removal from aqueous solutions	Synthesis, Characterization and Evaluation of Thiocarbazide Functionalization	[254]
Electro-deionization (EDI)	Th removal from aqueous solutions by electro-deionization (EDI)	Use of Response Surface Methodology for Optimization of Thorium (IV)	[255]

**Table 7:** Recent materials and processes for thorium recovery.

Processes	Applications	Characteristics	Refs.
Solvent extraction and separation of thorium (IV)	Separation of thorium	From chloride media by a Schiff base	[256]
Leaching and precipitation of thorium ions	Th separation from Cataclastic rocks	Abu Rusheid Area, South Eastern Desert, Egypt	[257]
Ion exchange materials	Process for purification of 225Ac from thorium and radium radioisotopes	Evaluation of inorganic ion exchange materials	[258]
Adsorption	Thorium adsorption	Graphene oxide nanoribbons/manganese dioxide composite material	[259]
	Thorium adsorption	Oxidized biochar fibers derived from Luffa cylindrica sponges	[260]
	Sorption behavior of thorium (IV)	Activated bentonite	[261]
	Adsorption of thorium (IV) response surface modelling and optimization	Amorphous silica	[262]
	Th (IV) adsorption	Titanium tetrachloride-modified sodium bentonite	[263]
	Evaluation of single and simultaneous thorium and uranium sorption from water systems	Electrospun PVA/SA/PEO/HZSM5 nanofiber	[264]
Synthesis and characterization of poly(TRIM/VPA)-functionalized graphene oxide nanoribbon aerogel	Highly efficient capture of thorium (IV)	Th ions separation from aqueous solutions	[265]
Vinyl-functionalized silica aerogel-like monoliths	Selective separation of radioactive thorium	Thorium separation from monazite	[266]
Recyclable GO@chitosan-based magnetic nanocomposite	Selective removal of uranium	From an aqueous solution of mixed radionuclides of uranium, cesium and strontium	[267]
Study of kinetics, thermodynamics, and isotherms of Sr adsorption	Graphene oxide (GO) and (aminomethyl) phosphonic acid–graphene oxide (AMPA–GO)	Th ion separation	[268]
Bulk liquid membrane containing Alamine 336 as a carrier	Kinetic study of uranium transport	Selectivity of the transport	[269]
Continuous bulk liquid membrane technique	Thorium transport	Modeling and experimental validation	[270]
Kinetic and isotherm analyses using response surface methodology (RSM)	Thorium (IV) adsorptive removal from aqueous solutions	By modified magnetite nanoparticles	[271]

## Data Availability

Data are contained within the article.

## Abbreviations

*AAS*	*Atomic Absorption Spectrometry*
*BLM (MLV)*	*Bulk Liquid Membrane*
*CME*	*Chemically Modified Electrode*
*CV*	*Cyclic Voltammetry*
*D*	*Dialysis*
*DM*	*Membrane Distillation*
*E*	*Extraction*
*ED*	*Electrodialysis*
*EDI*	*Electro-deionization*
*ELM*	*Emulsion Liquid Membrane*
*F*	*Filtration*
*G*	*Grinding*
*HFLM*	*Hollow Fiber Liquid Membrane*
*HLM*	*Hybrid Liquid Membrane*
*ICP-AES*	*Inductively Coupled Plasma–Atomic Emission Spectrometry*
*ICP-MS*	*Inductively Coupled Plasma–Mass Spectrometry*
*ICP-OES*	*Inductively Coupled Plasma–Optical Emission Spectrometry*
*IE*	*Ion Exchange*
*M*	*Milling*
*MF*	*Microfiltration*
*MOF*	*Metal-Organic Framework*
*MUF*	*Micellar Ultra-Filtration system*
*N*	*Neutralization*
*NF*	*Nanofiltration*
*P*	*Precipitation*
*PV*	*Pervaporation*
*RE*	*Re-Extraction*
*REE*	*Rare Earth Element*
*RO*	*Reverse Osmosis*
*S*	*Striping*
*SG*	*Gas Separation*
*TBP*	*Tri-Butyl Phosphate*
*UF*	*Ultrafiltration*
